# Chromosomal *qnrB19*-carrying *Escherichia coli* isolated from the stool sample of a community resident in Ecuador

**DOI:** 10.1128/mra.00046-24

**Published:** 2024-05-22

**Authors:** Hoa Thi Thanh Hoang, Mayumi Yamamoto, Manuel Calvopina, Carlos Bastidas-Caldes, Yoshimasa Yamamoto

**Affiliations:** 1 United Graduate School of Drug Discovery and Medical Information Sciences, Gifu University, Gifu, Japan; 2 Health Administration Center, Gifu University, Gifu, Japan; 3 One Health Research Group, Universidad De Las Americas, Quito, Ecuador; California State University San Marcos, San Marcos, California, USA

**Keywords:** quinolone resistance gene, *qnrB19*, chromosome, *Escherichia coli*

## Abstract

We identified a chromosomal *qnrB19* gene within a transposon in a colistin-resistant *Escherichia coli* strain isolated from the stool sample of an Ecuadorian resident. This finding suggests a more stable acquisition of quinolone resistance on chromosomes than that on plasmids and the potential for propagation to other DNA structures.

## ANNOUNCEMENT

Quinolones, recognized for their broad-spectrum antibiotic properties, are frequently used to treat various infections (1, 2). The primary resistance mechanism to these antibiotics often involves mutations in chromosomal genes of *Escherichia coli*, such as *gyrA* and *parC*, and to a less extent in *gyrB* or *parE* (3). The emergence of plasmid-mediated quinolone resistance (PMQR), known for its global spread (4) and ability to enhance resistance when combined with chromosomal mutations (5), is a growing concern. Notably, the transfer of resistance genes to the *E. coli* chromosome has been documented (6, 7); however, reports on the chromosomal transfer of PMQR genes are limited. Stabilization of resistance through such transfers is important for perpetuating quinolone resistance.

Here, we examined the LR-50 strain isolated from the stool sample of a resident of Santo Domingo, Ecuador, in 2019 (8). The strain was isolated at 37°C for 24 h using CHROMagar COL-APSE (CHROMagar, Pris, France), a selective agar medium for isolating colistin-resistant Gram-negative bacteria. The strain displayed ciprofloxacin sensitivity and moderate nalidixic acid resistance.

We extracted DNA from the LR-50 strain enriched from a single colony in LB broth, using the NucleoBond HMW DNA kit (Macherey-Nagel, Düren, Germany). Short-read sequencing of the extracted DNA was performed by Genome-Lead Co. (Kagawa, Japan) using DNBSEQ-G400RS (MGI Tech, Shenzhen, China), and long-read sequencing was performed using MinION Mk1C with an R9.4.1 flow cell (Oxford Nanopore Technologies, London, UK). A library of MinION- and DNBSEQ-sequenced data was prepared using a Rapid Barcoding kit (Oxford) and MGIEasy FS PCR-Free DNA Library Prep Set (MGI), respectively. The MinION library was prepared without the fragmentation step and then cleaned up with AMPure beads.

Regarding short-read sequencing data, the number of total reads was 5,834,336; the sequence lengths ranged from 17 to 149 bp. For the Nanopore long-read sequencing data, the number of total reads was 183,230; the read length N50 was 9,626 bp. Quality checks were performed using FastQC 0.11.9 and NanoPlot (9). A hybrid assembly was achieved using Unicycler 0.4.8 (10) with default settings. The final genome was polished using Pilon v.1.23 (11) and annotated using the DFAST v.1.6.0 (12) and RAST databases (13).

**Fig 1:**
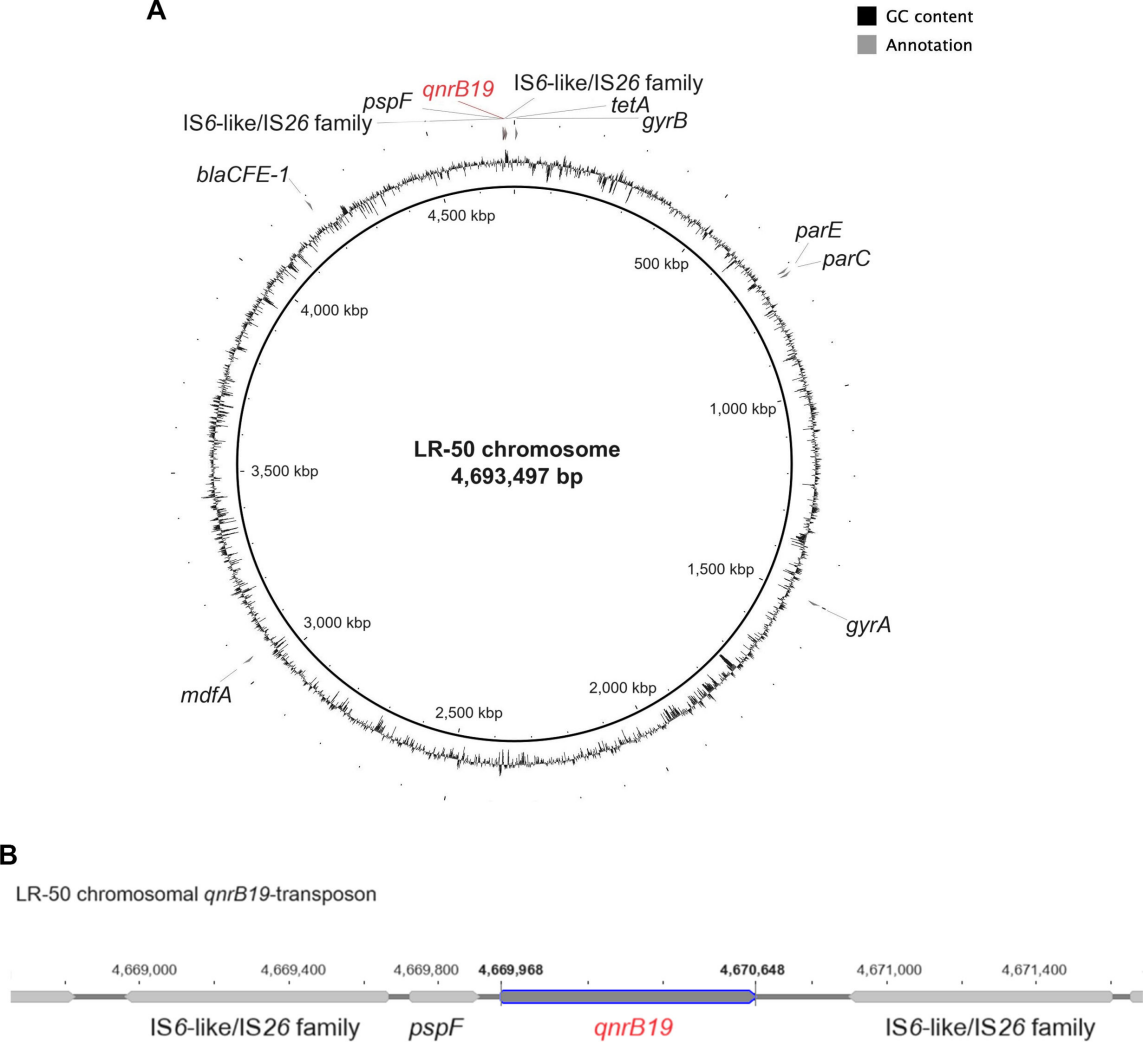
(**A**) The chromosomal distribution and locations of different genes in the LR-50 strain were displayed using BLAST Ring Image Generator 0.95 (http://brig.sourceforge.net). (**B**) The structure of the LR-50 chromosomal *qnrB19* transposon was analyzed using Geneious Prime 2021.2 software (https://www.geneious.com).

The LR-50 chromosome measured 4,693,497 bp with a G+C content of 50.8% (Fig. 1A) and was a circular double-stranded DNA as determined with a bridged assembly graph to complete the assembly using Unicycler. The number of CDSs was 4,363 with a coding ratio of 87.6% as determined using DFAST. The identified *qnrB19* gene is known to induce non-classical quinolone resistance (14). Analysis using the ResFinder 4.1 database revealed no chromosomal point mutations in quinolone resistance-related genes, including *gyrA*, *gyrB*, *parC*, and *parE*.

Interestingly, *qnrB19* in LR-50 is a part of a transposon structure flanked by IS6-like/IS26 family insertion sequences and a *pspF* operon transcriptional activator, which were detected by combining the annotations of DFAST and RAST (Fig. 1B). These insertion sequences, often linked to the spread of multidrug resistance, play a crucial role in the mobility of resistance genes (15). A recent study has reported the co-occurrence of *pspF* with *qnrB19* (16). The identification of PMQR genes on the chromosome in this study is a crucial insight for investigating quinolone resistance.

## Data Availability

The chromosome nucleotide sequence of *E. coli* LR-50 has been deposited in DDBJ/ENA/GenBank under accession number AP025551. The raw reads are available under DRR Run accession numbers DRR506514 and DRR506515. The annotation of the *E. coli* LR-50 chromosome by RAST is also available on figshare at LR-50_chromosome-RAST.
